# Viscoelastic properties of the Achilles tendon in vivo

**DOI:** 10.1186/2193-1801-2-212

**Published:** 2013-05-08

**Authors:** Jussi Peltonen, Neil J Cronin, Lauri Stenroth, Taija Finni, Janne Avela

**Affiliations:** Department of Biology of Physical Activity, Neuromuscular Research Center, University of Jyväskylä, Jyväskylä, Finland

**Keywords:** Stiffness, Hysteresis, Ultrasonography, Loading rate dependency, Medial gastrocnemius

## Abstract

It has been postulated that human tendons are viscoelastic and their mechanical properties time-dependent. Although Achilles tendon (AT) mechanics are widely reported, there is no consensus about AT viscoelastic properties such as loading rate dependency or hysteresis, in vivo. AT force-elongation characteristics were determined from 14 subjects in an ankle dynamometer at different loading rates using motion capture assisted ultrasonography. AT stiffness and elongation were determined between 10 – 80% of maximum voluntary contraction (MVC) force at fast and slow loading rates. As subjects were unable to consistently match the target unloading rate in the slow condition, AT hysteresis was only calculated for the fast rate. There was a significant difference between the fast and the slow loading rates: 120 ± 6 vs. 21 ± 1% of MVC s^-1^ (mean ± standard error), respectively. However, neither stiffness (193 ± 18 N mm^-1^ vs. 207 ± 22 N mm^-1^) nor elongation at any force level (13.0 ± 1.2 mm vs. 14.3 ± 0.9 mm at 80% of MVC) were significantly different between the fast and slow loading rates. Tendon hysteresis at the fast rate was 5 ± 2%. As stiffness was not sensitive to loading rate and hysteresis was small, it was concluded that elastic properties prevail over viscous properties in the human AT. The current results support the idea that AT stiffness is independent of loading rate.

**Electronic supplementary material:**

The online version of this article (doi:10.1186/2193-1801-2-212) contains supplementary material, which is available to authorized users.

## Introduction

Tendon stiffness is the most frequently used parameter to study tendon adaptation due to maturation (Waugh et al. 2011), aging (Narici and Maganaris. 2006), training (Foure et al. 2010;Westh et al. 2008), inactivity (Reeves et al. 2005) and disease (Zhao et al. 2009). However, it has been postulated that human tendons are viscoelastic and that their mechanical properties, like stiffness, depend on the rate at which the load is applied (Fung. 1993;Pioletti et al. 1998;Sanjeevi. 1982). This study was motivated by the observation that in the published literature, there is currently no consensus about the loading rate dependency or hysteresis of human tendons *in vivo*. In addition, viscoelastic behaviour is often disregarded in tendon adaptation studies and mechanical properties are determined at a variety of different loading rates.

In tendons that undergo substantial length changes during movement, like the Achilles tendon (AT), stiffness does not only determine functional properties of the tendon, but also the operational range of its muscle fibers (Fukunaga et al. 2001). Thus, stiffness is an important parameter that influences the whole muscle-tendon unit function. Although the idea of loading rate dependent stiffness has been experimentally refuted in vitro in both human (Wren et al. 2001b) and animal (Ker. 1981;Wang et al. 1995) tendons, it has been recently supported in vivo both in the AT (Gerus et al. 2011) and the patella tendon (Pearson et al. 2007).

Low hysteresis is advantageous for most tendons, because they, unlike muscles, store considerable amounts of elastic energy, which can be utilised in propulsion (Alexander and Bennet-Clark. 1977). While both in vitro and in vivo studies agree that hysteresis is an evident property of tendons, there is no agreement about the amount of hysteresis. In vitro, tendon hysteresis has been shown to be small, only 5–10% (Eliasson et al. 2007;Ker. 1981;Riemersma and Schamhardt. 1985;Wang et al. 1995). In vivo, tendon hysteresis was only shown to be equally small (7%) in the portion of the AT that attaches to soleus muscle (Zhao et al. 2009), but much higher (24%) in the medial gastrocnemius (MG) (Farris et al. 2011b;Wang et al. 2012) and lateral gastrocnemius (Lichtwark and Wilson. 2005) tendon.

Stiffness can be determined from any tendon, but the AT is perhaps the most frequently explored due to its importance in human locomotion and easy accessibility with ultrasound (US) due to its superficiality and pennated muscle fibres. In the current study, the AT was loaded in a controlled laboratory environment to accurately quantify its viscoelastic properties. The current results are particularly useful to interpret the results of AT adaptation studies; are changes in AT stiffness due to adaptation or due to a variety of different loading rates? The current study will also elucidate the relationship between elastic and viscous properties of the human AT. The study examined the following two research questions:

1. Do AT stiffness and elongation in vivo depend on the rate at which the load is applied?
2. Is AT hysteresis in vivo within the 5 to 10% range that has been commonly reported in vitro?

## Materials and methods

### Subjects

14 subjects (10 males and 4 females) participated in the study. Their age, height and mass were 36 ± 13 years, 173 ± 11 cm and 67 ± 11 kg (mean ± standard deviation), respectively. All subjects had a life-long training background in physical activities such as ball games and endurance running. Participants were informed about the procedures, benefits and possible risks involved in the study, and they all signed a written consent prior to the study. All methods were approved by the local ethical committee and the study conformed to the standards set by the latest revision of the Declaration of Helsinki.

### Protocol

Tendon mechanical properties were assessed in an ankle dynamometer where subjects voluntarily contracted and relaxed their calf muscles to stretch and release the AT. Contractions were performed at two distinctive speeds – fast and slow – to impose the AT to loading rates that are typically used in tendon adaptation studies. Prior to measurements, maximum voluntary contraction (MVC) force was determined as the best out of three trials. Then each subject performed 10 warm-up/practise contractions to stabilise the AT (Maganaris. 2003) and to learn the following characteristics of the trials: target force, which was 80% of MVC for both contractions speeds; and the target duration, which was either 1or 7 seconds for fast and slow contractions, respectively. Duration included both stretching and releasing of the tendon. Subjects received visual feedback of their force production through the monitor in front of them to help them to reach the target force level and durations. Six trials were recorded consisting of three trials for each speed. Trial order was randomised and a one-minute resting period was held between contractions to prevent muscle fatigue. Despite several attempts, subjects were not consistently able to control the release phase of the slow contraction. As a result, only the loading phase of the slow contraction is reported.

### Apparatus and collection of data

**Figure 1:**
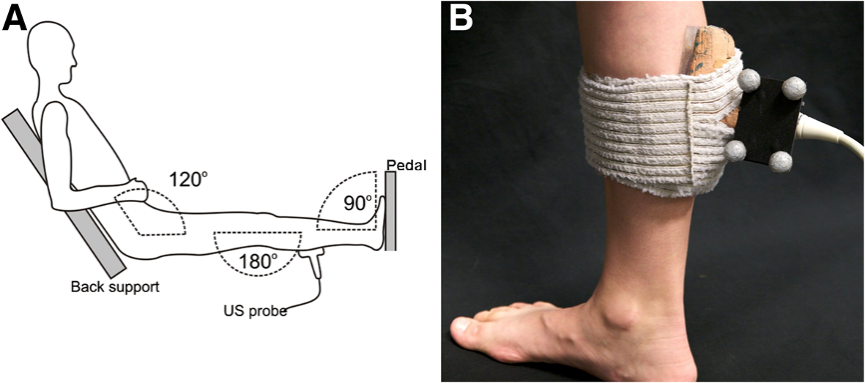
**A) Illustration of the ankle dynamometer and B) an ultrasound probe attached on the right leg.A**) Reproduced with permission from the Journal of Experimental Biology (Peltonen et al. 2010; doi:http://www.dx.doi.org/10.1242/jeb.033514). **B**) Reflective markers were placed on the ultrasound probe handle to enable motion analysis. The probe was attached in the sagittal plane over the myotendinous junction of the medial gastrocnemius muscle.

Tendon mechanical testing was performed in a custom-built ankle dynamometer (University of Jyväskylä, Finland). Subjects were seated with their knee extended, the ankle at a right angle (sole of the foot perpendicular to the shank) and the hip flexed to 60° (Figure 1A). Their right leg was tightly anchored between the back rest and the foot pedal where a force transducer (Precision TB5-C1, Raute, Nastola, Finland) was installed. Possible heel movement was quantified with a potentiometer placed under the heel. Foot reaction force and the heel position were collected with a 16-bit AD-board (CED 1401, Cambridge Electronic Design, England) at 1 kHz and stored on computer for later analysis.

AT length was measured using motion capture assisted ultrasonography (US). With this method, US image coordinates can be transformed to the laboratory coordinate system (CS) for accurate calculation of AT length. To identify the myotendinous junction (MTJ) of the medial gastrocnemius (MG) muscle, a linear array US probe (Aloka 5712, Osaka, Japan) was placed in the sagittal plane over the right leg 2 cm medial to the junction separating the medial and lateral portions of the gastrocnemius muscle. An impedance-matched acoustic pad was placed under the probe to ease propagation of ultrasound waves, and the probe was secured to the leg with elastic bandages (Figure 1B). A digital camera (InLine 250, Fastec Imaging, San Diego, USA) was placed on the ankle dynamometer’s left side to image movement of the probe during measurements. The camera’s optical axis was perpendicular to the sagittal plane and the camera was focused on the US probe’s four reflective markers that were placed on the probe handle to enable movement tracking. The camera and the US unit operated at 125 frames s^-1^ for the fast loading rate and 60 frames s^-1^ for the slow loading rate. The data collection rate was reduced for the slow condition due to memory limitation in the US imaging unit (Aloka Alpha 10, Osaka, Japan). Because the loading rate was several times slower at the slow rate, the lower sampling frequency was also adequate. US images were stored to the imaging unit and the digital camera’s video was recorded to computer for later export. The camera and US images were synchronised with a square-wave pulse that was fed into the US unit’s digital input and used to trigger a flashing light signal visible on the video.

### Analysis and statistics

AT force was calculated by multiplying the pedal reaction force with a gear ratio (foot lever arm divided by tendon lever arm). Foot lever arm was determined as the distance between the first metatarsal and the centre of the medial malleolus and tendon lever arm as the distance between the medial malleolus and the posterior surface of the calcaneus (Peltonen et al. 2010,2012). Lever arm lengths were taken as a projection along the sole of the foot when it was perpendicular to the shank. Therefore, lever arms were considered to be perpendicular to the line of force.

**Figure 2:**
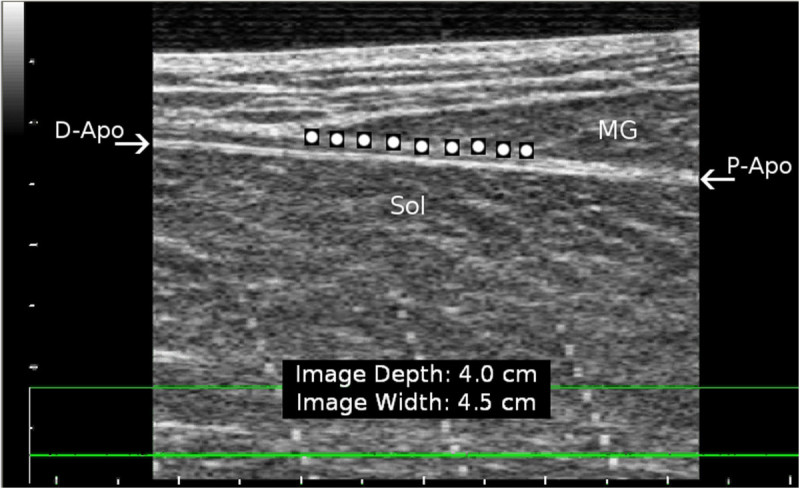
**Placement of the tracking markers over the region of interest in the ultrasound image.** Nine markers were placed on medial gastrocnemius (MG) muscle along the aponeurosis separating the soleus (Sol) and MG muscles. D-Apo = distal aponeurosis; P-Apo = proximal aponeurosis.

AT length was calculated as the distance between the MTJ of the MG muscle and the bony superior and posterior surface of the heel. Initial position of the heel was taken when the foot was properly installed to the pedal. Possible heel displacement in the superior-inferior direction during subsequent muscle contraction was measured with a position sensor located under the heel. MTJ displacement was analysed from the US images with software that exploits pyramidal implementation of the Lukas-Kanade feature tracking (Bouguet, 2000, May). The software requires the user to place nine tracking points over the area of interest as shown in Figure 2. The tracking points were placed just superior to the MTJ along the aponeurosis separating MG and soleus, but still on the side of MG. We have found this placement to yield the most repeatable results. The tracking algorithm has been previously shown to be accurate and repeatable (Magnusson et al.^2003^). After tracking, MTJ coordinates of each trial were transformed from the US probe CS to the laboratory CS, which was determined by a stationary video calibration object. The moving CS of the US probe was defined by the four reflective markers placed on the probe handle (Figure 1B). The origin of the US image relative to the probe markers was determined by placing an echogenic marker on the image origin and measuring its distance from the markers. Two dimensional analysis was considered adequate because all movement could be restricted to the sagittal plane (Peltonen et al. 2010). A similar procedure has also been used in space (Gerus et al. 2011;Lichtwark and Wilson. 2005). Tendon elongation was calculated by subtracting initial tendon length at the onset of force production, and strain was calculated by dividing elongation by initial length.

AT force-elongation curves were normalised in time and then averaged resulting in one curve per subject per loading rate. The mean coefficients of variation between each trial for the same subject and the same loading rate were 6% and 7% for the durations of the fast loading and unloading phase, respectively, and 9% for the duration of the slow loading phase. All 14 subjects were included in the analysis of the fast contraction and 10 in the analysis of the slow contraction. Reasons for exclusion were: data were corrupted (1 case); duration of the loading phase deviated by more than 40% of the target duration (1 case); failure to reach the target force level (2 cases).

**Figure 3:**
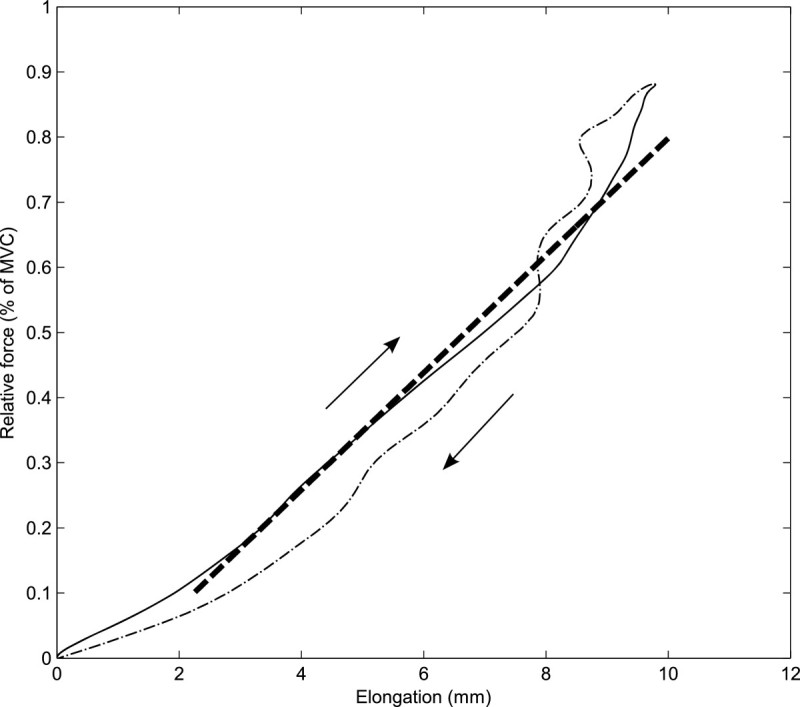
**Example of tendon stiffness and hysteresis deduction from the load-deformation curve.** Stiffness (broken thick line) was calculated as the slope of the ascending limb (solid line) of the force-elongation curve between 10-80% of MVC. Hysteresis was calculated as the area between the ascending (solid line) and descending limb (dash-dot line). MVC = maximum voluntary contraction.

AT stiffness was calculated as the slope of the least-squares line of the ascending limb of the force-elongation curve between 10% and 80% of MVC force. Loading and strain rates were calculated as the slope of the force-time and strain-time curves, respectively. Hysteresis was calculated by subtracting the area under the descending limb of the force-elongation curve from the area under the ascending limb and dividing the difference by the area under the ascending limb (Figure 3).

**Figure 4:**
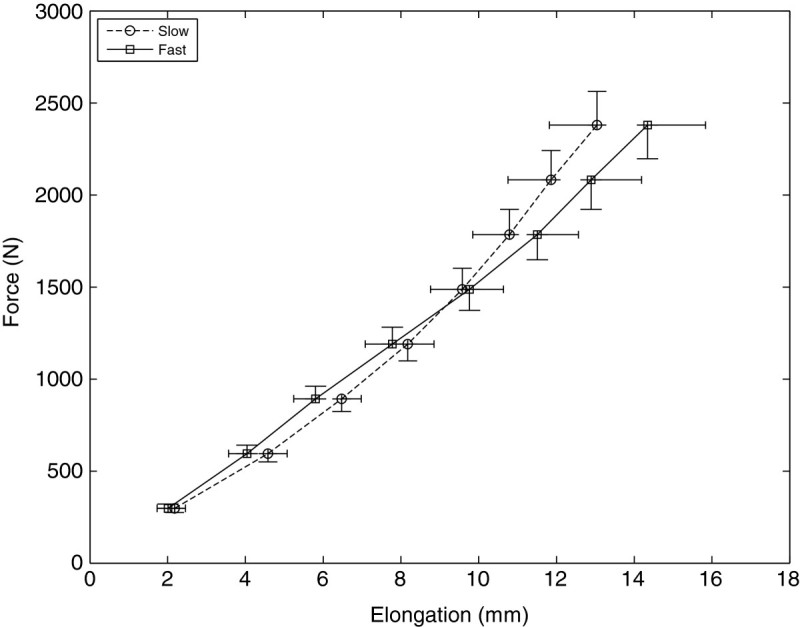
**Average Achilles tendon force-elongation curves at the fast and slow loading rates.** The data points are given at 10% intervals ranging from 10–80% of maximum voluntary force. The solid line and square markers indicate the fast rate, and the broken line and circle markers indicate the slow rate. Elongation was not significantly different between the fast and slow rates at any force level.

Loading phases of individual force-elongation curves were further averaged to yield separate loading curves for the slow and fast contractions (Figure 4). Averaging was done at 10% intervals ranging from 10% to 80% of MVC and included only those subjects that had data for both loading rates (N = 10).

Wilcoxon’s signed-rank test was used to test differences in stiffness and elongation between the fast and slow contractions. Linearity of average force elongation curves was tested with a least squares linear regression method. The level of significance was always set to P < 0.05. Errors in figures and tables are the standard error (SE) of the mean.

## Results

Table 1 displays AT loading rate sensitivity and hysteresis. The average fast loading rate was 120 ± 6% of MVC s^-1^ (mean ± SE) and it was significantly higher than the average slow loading rate, 21 ± 1% of MVC s^-1^ (N = 10, P < 0.05). However, AT stiffness did not change between the fast and slow loading rates being 193 ± 22 N mm^-1^ and 207 ± 18 N mm^-1^, respectively (N = 10, P = 0.105).

**Table 1:** **Achilles tendon loading rate sensitivity and hysteresis**

Subject	Sex	Loading rate (% of MVC s^-1^)		Stiffness (N mm^-1^)		Hysteresis (%)
		Slow	Fast	Slow	Fast	Fast
1	M	18	91	300	267	-7
2	M	20	151	255	310	15
3	M	20	126	191	168	-1
4	F	20	108	196	179	-4
5	F	23	126	273	241	-1
6	M	19	125	156	124	21
7	M	19	97	247	253	6
8	M	23	108	176	154	6
9	F	21	118	144	119	13
10	M	27	153	132	120	-4
11	M		144		227	-4
12	M		180		188	9
13	F		111		132	6
14	M		264		279	13
mean (subjects 1-10)		21	120	207	193	4
SE (subjects 1-10)		1	6	18	22	3
mean (all)			136		197	5
SE (all)			12		17	2

*MVC* maximum voluntary contraction, *SE* standard error.

Tendon hysteresis was determined for all subjects at the fast rate (Table 1). Hysteresis was on average 5 ± 2% (N = 14) and it ranged from −7% to 21%. There was no difference between the mean loading time (0.589 s) and the mean unloading time (0.588 s) at the fast rate (N = 14, P = 0.890). The possible relationship between hysteresis and the difference in loading and unloading time was also tested, but the correlation coefficient proved to be non-significant (N = 14, R = −0,425, P = 0.129). It must be noted here that negative hysteresis means that the tendon released more energy than it originally stored, which is not possible. This issue is addressed further in the discussion.

Figure 4 shows average AT elongations at 10% intervals of MVC. At the maximum measured force level, which was 80% of MVC, the AT elongation was 14.3 ± 1.2 mm at the fast rate and 13.0 ± 0.9 mm at the slow rate, but the difference was not statistically significant (N = 10, P = 0.064). The same was true for all force levels: there were no statistical differences in AT elongation.

## Discussion

The major findings of the current study were that: 1) AT stiffness and elongation were independent of the loading rate and 2) AT hysteresis was small (5% on average). The current data are in line with the majority of previous in vitro tendon studies indicating time-independent mechanical behaviour (Abrahams. 1967;Ker. 1981;Mabuchi et al. 1991;Noyes et al. 1974;Wang et al. 1995;Woo et al. 1990;Wren et al. 2001b) and small hysteresis (Bennett et al. 1986;Eliasson et al. 2007;Ker. 1981;Riemersma and Schamhardt. 1985;Wang et al. 1995).

### Influence of tendon material

Early failure experiments demonstrated that ligaments were more prone to ruptures at high strain rates than at low strain rates (Crowninshield and Pope. 1976;Noyes et al. 1974). Because later studies did not find such a relationship (Danto and Woo. 1993;Ng et al. 2004;Woo et al. 1990;Wren et al. [Bibr CR45][Bibr CR46]), it has been postulated that tendon viscoelastic properties may vary (Wren et al. 2001b). The finding that high-stressed flexor tendons have a lower hysteresis than low-stressed extensor tendons supports this idea (Shadwick. 1990). Because high-stressed tendons have also been shown to have higher fatigue resistance than low-stressed tendons (Pike et al. 2000), it has been postulated that high-stressed tendons are made of different material, possibly to compensate for a lower safety factor (Ker et al. 2000). In humans, the AT has to withstand stresses around 80 MPa (Lichtwark and Wilson. 2005) and can be categorised as a high-stressed tendon. In comparison, maximum stress in the patella tendon may be only half of that, 40 MPa (Hansen et al. 2006). Thus, functional requirements may explain why the patella tendon and the AT are made of different material and why the patella tendon exhibits loading rate sensitivity (Pearson et al. 2007), whereas the AT in the current study did not.

All subjects in the current study had a history of regularly stressing their AT’s in activities that require cyclic stretching and releasing of the AT, which could explain their rather non-viscous properties. The exact mechanism of tendon mechanical adaptation is not understood, but there is substantial evidence showing that tendon stiffness increases during maturation (Waugh et al. 2011) and training (Westh et al. 2008), and decreases during aging (Narici and Maganaris. 2006) and inactivity (Reeves et al. 2005). However, there is usually no clear evidence of causality; for example, an age-related decrease in stiffness may be a consequence of the aging process, but it may also be caused by decreased physical activity and loss of muscle force, therefore suggesting that tendon adapts to usage (Stenroth et al. 2012). Alterations in tendon hysteresis are much less frequently reported than changes in stiffness. However, there is some evidence to suggest that AT hysteresis could decrease after plyometric training (Foure et al. 2010) without any significant change in AT cross-sectional area. This supports the adaptation to usage theory and indicates that by regularly engaging in physical activities, the current subjects may have achieved or maintained an AT that has a small hysteresis and is suitable for efficient elastic energy storage and return.

### Implications for human movement

Low hysteresis is advantageous for tendon not only because it enables high elastic energy return but also because it minimises heat damage (Alexander. 2002). This applies to walking, running and jumping, during which high efficiency is required. On the contrary, during landing, where mechanical energy is absorbed rather than stored and returned, high hysteresis could be useful. This is probably unnecessary because tendon acts as an energy buffer by rapidly stretching and storing energy that is then slowly absorbed during subsequent active muscle lengthening. This substantially lowers energy absorption rate as well as muscle eccentric contraction velocity – as compared to fast muscle stretch – and supposedly protects against muscle damage (Konow et al. 2011). It is difficult to imagine how varying stiffness could improve this ability. Higher stiffness would obviously result in lower tendon strain. However, reducing tendon strain also reduces strain energy storage and may compromise the tendon’s ability to act as an energy buffer. Thus, it seems plausible that tendon strains are proportional to the load and independent of the loading rate.

In the current study, the mean slow strain rate was 1.7% s^-1^ and the mean fast strain rate was 10% s^-1^. Even faster strain rates are achieved in running (15 – 70% s^-1^)(Farris et al. 2011a;Lichtwark et al. 2007), but the current strain rate range overlaps with that of walking (Lichtwark et al. 2007). The quite substantial strain rate range (6-fold difference), without any noticeable changes in tendon stiffness or elongation, indicates that AT mechanical behaviour may be strain rate independent throughout the physiological strain rate range. Regardless of whether the current results can be generalised to full speed motion or not, they provide evidence that the strain rates that are typically used in AT mechanical testing do not affect its stiffness or elongation.

### Methodological considerations

In the published literature, AT elongation has been determined with several methods. The challenge has been to take into account the displacements of the myotendinous junction, the heel and the US probe. Sometimes these parameters have been estimated, but this could lead to overestimation of AT elongation by 40% (Gerus et al. 2011). The current study does not suffer from this limitation. The AT elongation was calculated by measuring both heel movement and MTU displacement within the same contraction, without any corrections, with a method that combines ultrasonography and motion analysis. We consider this method to be the most accurate to date. Because AT force was estimated, it is influenced by variation in moment arm lengths. We have verified with pressure insoles that the point of application of force moves slightly towards the 1^st^ metatarsal as the force increases, thus increasing the foot lever arm length. However, the heel lever arm length increases as well (Rugg et al. 1990) and may counterbalance this effect. Therefore, we have used a constant gear ratio in our calculations.

The current mean force-elongation curves (Figure 4) were rather linear: Pearson’s linear correlation coefficients were R = 0.998 and R = 0.999 for the fast and slow loading rates, respectively. Low stiffness, or toe region, was either absent or small, as indicated by the intercept of the linear regression that was not significantly different from zero at the fast rate (student’s t-test; P = 0.095) and only slightly different from zero at the slow rate (P = 0.037). The lack of toe region was probably due to the initial tension (approximately 0.3 kN) acting on the AT at the onset of muscle contraction. Due to the initial tension, possible fiber slack is removed and tendon is stretched within the linear region from the beginning of the contraction. This explains why the current average hysteresis is low (5%) and comparable to those achieved during mechanical testing of isolated tendons (Ker. 1981).

In the current study, the range in AT hysteresis (Table 1) was 28% (from −7% to 21%). No single reason for this variation has been yet identified, but it is typical when determining tendon hysteresis in vivo. Previously reported ranges include 43% during running (from 2% to 45%) (Farris et al. 2011b) and 24% during one-legged hopping (from 15% to 39%) (Lichtwark and Wilson. 2005). In viscous material, variations in hysteresis could be induced by alternating loading and unloading rates. However, this idea was refuted in the current study, because there was no difference between the fast loading and unloading times. We have recently suggested that variation in hysteresis can be attributed to signal desynchronisation and differentiating synergist muscle activation (Finni et al. 2012). For example, if the total duration of the loading and unloading phases is 1 second, and the sampling rate is 100 Hz, desynchronisation of 1 frame may under- or overestimate hysteresis by 10% (Finni et al. 2012). There are methods to overcome this problem and signal averaging is one of them: Averaging force-elongation curves over several trials reduces the effect of random desynchronisation by one frame. The more compelling challenge is the anatomy of the tendon, because the AT is a common tendon of the three heads of the triceps surae muscles, and each head can stretch the AT independently causing regional strain variations (Finni et al. 2006). This may cause random and systematic errors. While random errors are treated by averaging, systematic errors are harder to detect. However, their influence on conclusions can be minimized by having the same settings, e.g. joint angles, for both the fast and the slow rate. In summary, measurement uncertainties are an inevitable consequence of determining human AT hysteresis in vivo. As a result, experimentally deduced hysteresis may also include negative values if the mean hysteresis is low (less than 10% as indicated by isolated tendons). Considering negative hysteresis values to be erroneous and rejecting them may lead to biased results (Finni et al. 2012). This could also explain why reported values for hysteresis are typically higher in vivo than in vitro.

## Conclusion

In the AT of physically active individuals, elastic properties prevail over viscous properties. Thus, the AT is well suited to elastic energy storage and return during human walking, running and jumping. The current results are in agreement with the majority of in vitro studies that indicate rather non-viscous properties in tendons across species. However, the findings about tendon hysteresis remain inconclusive. In vitro studies typically demonstrate low tendon hysteresis (<10%), whereas in vivo studies have often shown higher hysteresis (>20%). Our results support the former, which we believe represent more physiological values. It is postulated that the differences in hysteresis in living tendons could be attributed to methodological variations as well as material differences in tendons induced by physical activity backgrounds and/or tendon anatomical location and function. The current results support the idea that tendon stiffness is not affected by the rate at which the load is applied during tendon mechanical testing in vivo.

## Authors’ original submitted files for images

Below are the links to the authors’ original submitted files for images. Authors’ original file for figure 1 Authors’ original file for figure 2 Authors’ original file for figure 3 Authors’ original file for figure 4

## Abbreviations

AT: Achilles tendon
CS: Coordinate system
MG: Medial gastrocnemius
MTJ: Myotendinous junction
MVC: Maximum voluntary contraction
US: Ultrasonography.
