# Supplementary figures and images for: Viscoelastic properties of the Achilles tendon in vivo
Source: Springerplus. 2013 May 8;2(1):212. doi: 10.1186/2193-1801-2-212 (PMC3661039; doi:10.1186/2193-1801-2-212)

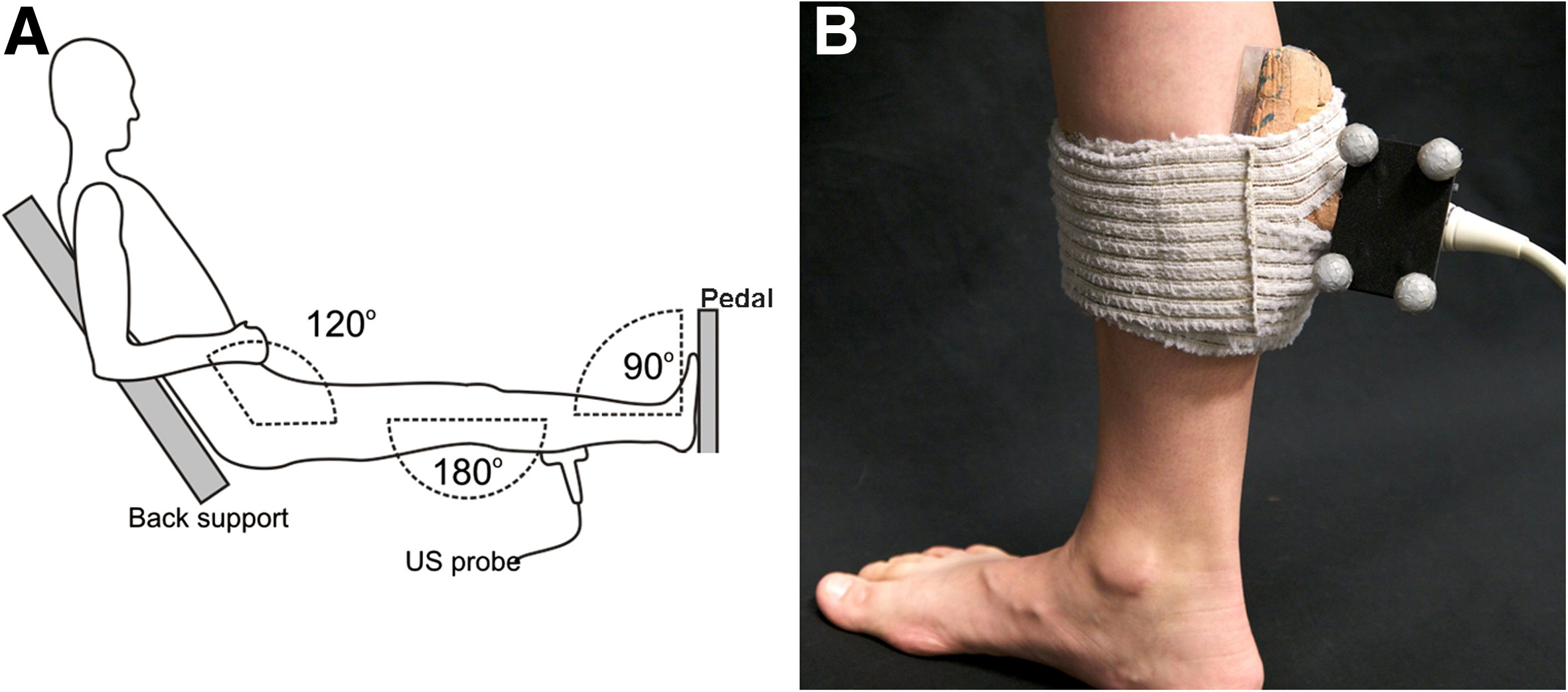

Supplement: Supplementary file 1 — Authors’ original file for figure 1 [file 40064_2013_271_MOESM1_ESM.jpeg]

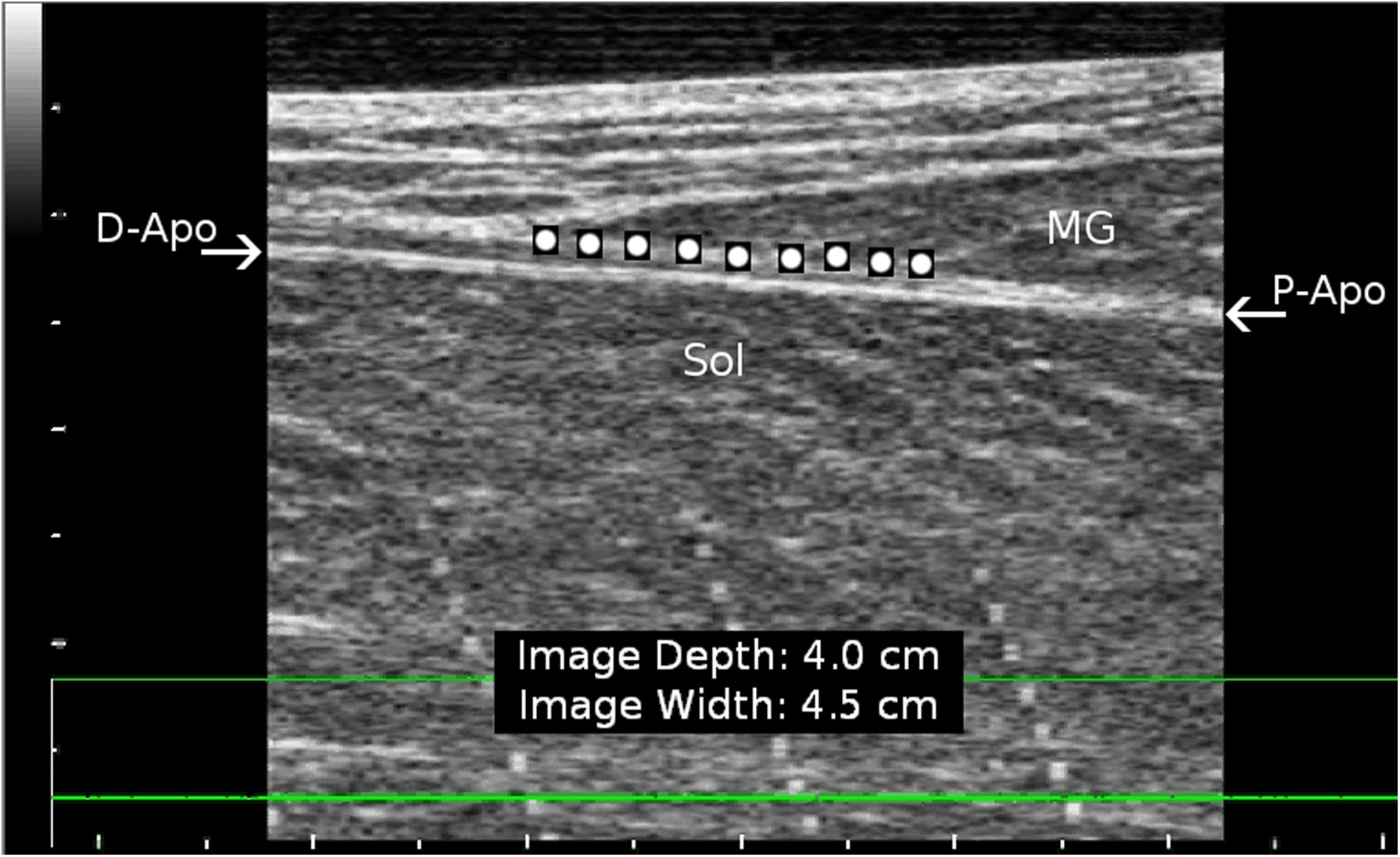

Supplement: Supplementary file 2 — Authors’ original file for figure 2 [file 40064_2013_271_MOESM2_ESM.jpeg]

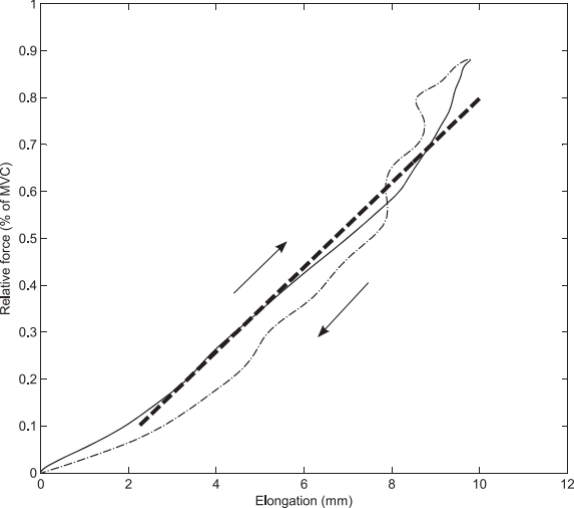

Supplement: Supplementary file 3 — Authors’ original file for figure 3 [file 40064_2013_271_MOESM3_ESM.pdf]

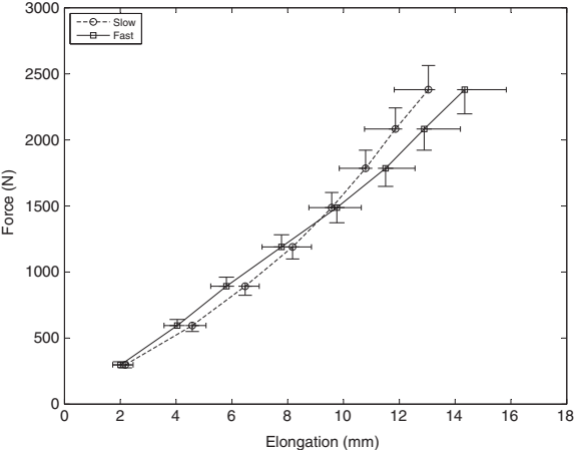

Supplement: Supplementary file 4 — Authors’ original file for figure 4 [file 40064_2013_271_MOESM4_ESM.pdf]
